# Treatment for radiographically active, sputum culture-negative pulmonary tuberculosis: A systematic review and meta-analysis

**DOI:** 10.1371/journal.pone.0293535

**Published:** 2023-11-16

**Authors:** Adam Thorburn Gray, Liana Macpherson, Ffion Carlin, Bianca Sossen, Alexandra S. Richards, Sandra V. Kik, Rein M. G. J. Houben, Peter MacPherson, Matteo Quartagno, Ewelina Rogozińska, Hanif Esmail

**Affiliations:** 1 Institute for Global Health, University College London, London, United Kingdom; 2 MRC Clinical Trials Unit at University College London, London, United Kingdom; 3 Infectious Diseases Unit, Liverpool Royal Hospitals NHS Foundation Trust, Liverpool, United Kingdom; 4 Department of Medicine, Faculty of Health Sciences, University of Cape Town, Cape Town, South Africa; 5 TB Modelling Group, TB Centre, London School of Hygiene and Tropical Medicine, London, United Kingdom; 6 Department of Infectious Disease Epidemiology, Faculty of Epidemiology and Public Health, London School of Hygiene and Tropical Medicine, London, United Kingdom; 7 FIND, The Global Alliance for Diagnostics, Geneva, Switzerland; 8 School of Health & Wellbeing, University of Glasgow, Glasgow, United Kingdom; 9 Clinical Research Department, London School of Hygiene and Tropical Medicine, London, United Kingdom; 10 Wellcome Centre for Infectious Diseases Research in Africa, Institute of Infectious Diseases and Molecular Medicine, University of Cape Town, Cape Town, South Africa; Shandong Public Health Clinical Center: Shandong Provincial Chest Hospital, CHINA

## Abstract

**Background:**

People with radiographic evidence for pulmonary tuberculosis (TB), but negative sputum cultures, have increased risk of developing culture-positive TB. Recent expansion of X-ray screening is leading to increased identification of this group. We set out to synthesise the evidence for treatment to prevent progression to culture-positive disease.

**Methods:**

We conducted a systematic review and meta-analysis. We searched for prospective trials evaluating the efficacy of TB regimens against placebo, observation, or alternative regimens, for the treatment of adults and children with radiographic evidence of TB but culture-negative respiratory samples. Databases were searched up to 18 Oct 2022. Study quality was assessed using ROB 2·0 and ROBINS-I. The primary outcome was progression to culture-positive TB. Meta-analysis with a random effects model was conducted to estimate pooled efficacy. This study was registered with PROSPERO (CRD42021248486).

**Findings:**

We included 13 trials (32,568 individuals) conducted between 1955 and 2018. Radiographic and bacteriological criteria for inclusion varied. 19·1% to 57·9% of participants with active x-ray changes and no treatment progressed to culture-positive disease. Progression was reduced with any treatment (6 studies, risk ratio [RR] 0·27, 95%CI 0·13–0·56), although multi-drug TB treatment (RR 0·11, 95%CI 0·05–0·23) was significantly more effective than isoniazid treatment (RR 0·63, 95%CI 0·35–1·13) (p = 0·0002).

**Interpretation:**

Multi-drug regimens were associated with significantly reduced risk of progression to TB disease for individuals with radiographically apparent, but culture-negative TB. However, most studies were old, conducted prior to the HIV epidemic and with outdated regimens. New clinical trials are required to identify the optimal treatment approach.

## Introduction

Globally in 2021, 2 million (37%) of the 5·3 million people notified with pulmonary tuberculosis (TB) were not bacteriologically confirmed. This percentage has remained unchanged over recent years [1] and is also found in high resource settings, such as the UK (39·3%) [2]. Lack of bacteriological confirmation may be due to technical factors including sample quality, laboratory processing, diagnostic test sensitivity [3], or clinical factors as some individuals have paucibacillary TB disease (e.g. children, people living with HIV, extra-pulmonary disease). For these reasons, inability to detect *Mycobacterium tuberculosis* does not exclude active disease and in some circumstances radiology, symptomatology, or histology, potentially combined with exposure history, are used to inform treatment decisions.

Active pulmonary TB is usually confirmed by a sputum bacteriological test (e.g. smear, molecular test, culture). However, disease pathology and infectiousness can precede symptom development; and these individuals with subclinical disease may be detectable radiographically [4]. This has long been recognised and in the mid-twentieth century mass chest X-ray (CXR) screening for TB was widely implemented. After the 1970s the cost effectiveness of the approach was questioned, especially in countries experiencing a rapid decline in incidence, and the practice was largely abandoned [5]. However, increasingly affordable and accessible technology (e.g. digital X-ray, computer-aided-detection software) has led to a re-expansion of CXR-based screening, and the potential utility in increasing case detection has been re-emphasised in the updated WHO TB screening guidance [6].

Although CXR alone cannot be used to confirm TB, the radiographic pattern can be highly suggestive in TB endemic settings. TB disease is dynamic and does not always progress and can undergo regression with subsequent fibrosis and calcification, radiographic features can therefore help to distinguish active disease (with features such as infiltration, consolidation, poorly defined nodules, and cavities) from inactive disease (with features such as discrete nodules and fibrotic scarring with or without volume loss or retraction), which affects prognosis [7]. In a recent meta-analysis we showed that in those who did not receive treatment, the risk of progression from bacteriologically negative to positive TB is approximately 10% per year in those with CXR changes suggestive of active TB, and 1% in those with changes suggestive of inactive TB [8].

Management of TB remains rooted in a binary approach: treat for so-called ‘active’ TB disease or ‘latent’ TB infection [9]. For drug-susceptible active TB in adults (excluding central nervous system disease) a one-size-fits-all strategy prevails irrespective of bacillary burden [6]. The standard 6-month treatment of rifampicin and isoniazid supplemented with pyrazinamide and ethambutol for the first two months was developed in trials designed to identify successful treatments for smear positive disease [10]. Hence, treatment duration and composition are driven by the requirements of the most extensive, multibacillary forms of disease, potentially over treating more paucibacillary states [9]. Recognising the false dichotomy of binary disease management, the recent SHINE trial has shown that minimal, largely bacteriologically negative, disease in children can be treated with a four-month regimen [11].

The optimal management for bacteriologically negative, radiographically active pulmonary TB in adults is unknown. These patients are less likely to be symptomatic at time of diagnosis and may be less willing to tolerate the duration, pill burden, and drug toxicity of the standard 6-month regimen [12]. Shorter, less toxic regimens could reduce adverse events, increase adherence, and reduce individual and programmatic costs, but clinical trials are needed to determine effectiveness. We sought to systematically identify evidence from clinical trials that compared the effectiveness of anti-tuberculous regimens to placebo, observation, or alternative regimens, on disease progression in people with bacteriologically negative, but radiographically apparent pulmonary TB.

## Materials and methods

### Search strategy and selection criteria

We conducted a systematic review and meta-analysis in accordance with the Preferred Reporting Items for Systematic Reviews and Meta-Analyses (PRISMA) [13]. This study was registered with PROSPERO (CRD42021248486). We searched EMBASE (Ovid from 1947), MEDLINE (Ovid from 1946), the Cochrane Infectious Diseases Group specialised register, and Web of Science (from 1900) with no language restrictions. Eligible studies were randomised and non-randomised prospective trials comparing treatment against placebo, observation, or alternative treatment, for the management of children and adults with suspected pulmonary TB based on CXR, but with negative respiratory tests (culture or molecular test); the search strategy is available (Supplementary Material). Initial searches were performed on 17^th^ March 2021 and updated on 18^th^ October 2022; abstracts and eligible full manuscripts were independently screened by two of LM, FC, or ATG, with discrepancies resolved by HE. References of included articles were reviewed for additional relevant articles.

### Data extraction and quality assessment

Summary data were independently extracted by two of LM, FC, or ATG, into a piloted database to inform a primary outcome of progression to bacteriologically confirmed pulmonary TB, defined as at least one positive smear, culture, or molecular test on sputum, laryngeal swab, or gastric lavage during follow up. Further data were extracted where available to inform secondary outcomes including clinical or radiographic evidence of progressive active TB at multiple time-points (0, 6, 12, 24, 48, 60 months); adverse events; drug resistance; treatment adherence; and loss to follow-up (Supplementary Table S1 in S1 File). Data on country-level TB incidence were extracted to inform baseline risk of TB disease if available. Two reviewers independently assessed risk of bias of included studies using recommended tools for randomised (RoB 2·0) [14] and non-randomised trials (ROBINS-I) [15].

### Data analysis

Clinical and epidemiological characteristics were summarised, including the number and percentage of participants excluded at baseline due to positive cultures and the duration of follow-up. To investigate the effect of treatment on progression to bacteriologically confirmed TB, we pooled data from comparable trials (active radiographic changes and use of an inactive comparator) using a random-effects model with restricted maximum likelihood (REML) estimation of variance method. The effects are reported as pooled unadjusted relative risks (RR) and number-needed-to-treat (NNT, i.e. the number of participants that would be required to be treated to avert one progression to active pulmonary TB) [16]. For cohorts with zero incident cases over the follow-up period, a fixed value was added. Heterogeneity was assessed by inspecting forest plots and by calculating the I^2^ statistic and explored using subgroup analyses: treatment regimen composition (multi-drug vs simple isoniazid-based; stratification determined after qualitative review of included studies); case finding methodology (active case-finding [ACF] vs passive case-finding [PCF]); number of baseline sputa samples (fewer than three vs three or more); HIV prevalence in participants (<1% vs >1%); and severity of baseline radiographic abnormality (single lobar disease vs more extensive disease). A funnel plot was constructed to assess small study effects and potential for publication bias. Sensitivity analyses were conducted using studies with only low or some risk of bias, using all TB diagnoses (with or without bacteriological confirmation) for the primary outcome, and using per-protocol results (assessing those that completed study follow up). To compare different duration of regimens we performed a network meta-analysis within the frequentist framework and a ‘contrast-based’ model, for the main outcome using studies with comparable populations (input data available in Supplementary Table S10 in S1 File). As the network was sparse, we fitted a fixed-effects model to produce relative treatment rankings [17]. Analyses were performed using R (Version 4·2·2, R Foundation for Statistical Computing, Vienna) using the metafor package v3·8–1 and Stata (version 17·0, Statacorp, College Station, Texas, USA) using network package [18, 19].

## Results

**Fig 1:**
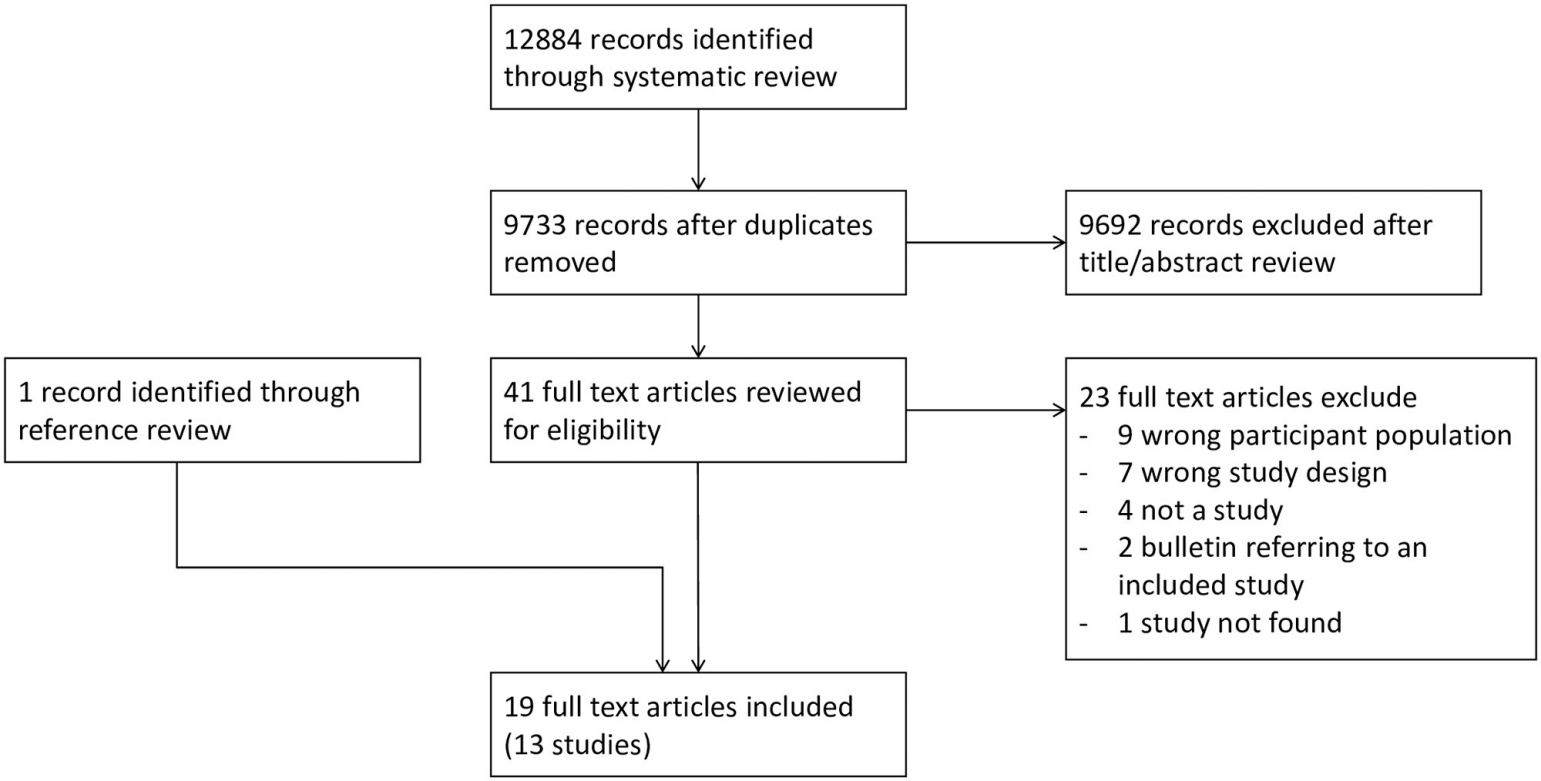
Study selection flow diagram.

We identified 9733 unique publications for title and abstract screening of which 41 met criteria for full text review. Of these 18 were included, with one additional publication identified through reference screening, resulting in 19 manuscripts relating to 13 studies (Fig 1 and Supplementary Material). Studies excluded at full text review are listed with rationale for exclusion (Supplementary Table S2 in S1 File).

The studies, 12 of which were randomised controlled trials, were conducted between 1955 and 2018 across multiple WHO regions (African, European, South-East Asia, and Western Pacific) and had similar eligibility criteria (Table 1 and Supplementary Table S3 in S1 File). Six studies recruited participants using ACF, with the remainder recruiting participants who had presented independently to healthcare services (e.g. PCF). There was heterogeneity in the number of negative sputum cultures required at baseline (ranging from 1 to 6 samples), with only Turkova *et al*. using molecular tests to define participants as microbiologically negative. There was heterogeneity in imaging modality used (e.g. plain CXR, miniature film) and methodology for CXR classification, with only two studies using predefined criterion (Supplementary Table S4 in S1 File). Most studies compared treatment against placebo or observation, with three of the more recent studies comparing different regimens. There was limited information on medicine administration; four studies reported on adherence.

**Table 1:** Study characteristics.

	Setting and case finding methodology	Country	Baseline sputum culture-positive (excluded)	Number recruited	TB disease activity* at baseline CXR	Control Arm	Intervention Arm	Primary outcome
Clayson (1963) [33]	Not reported (ACF)	Scotland	22/219 (10%)	219	Mixed	No treatment	6HPAS**	Radiological or bacteriological diagnosis of TB at 5 years
Frimodt-Moller (1960) [36]	Community CXR survey (ACF)	India	32/468 (6·8%)	468	Active	Placebo	3H (+-PAS) 6H (+-PAS) 12H (+-PAS)	Bacteriological diagnosis of TB at 4 years
Pamra (1971) [39]	Community CXR survey (ACF)	India	22/424 (5·2%)	424	Mixed	Placebo	12H	Radiological or bacteriological diagnosis of TB at 6 years
Aneja (1979) [38]	Symptomatic patients presenting to care (PCF)	India	144/457 (31·5%)	457	Mixed	Placebo	12HTh	Radiological or bacteriological diagnosis of TB at 1 year
Thompson (1982) [31]	Annual CXR surveillance (ACF)	Multiple (Europe)	Not reported	27830	Inactive	Placebo	3H 6H 12H	Bacteriological diagnosis of TB at 5 years
Anon (1984) [40]	Adults presenting to care (PCF)	Hong Kong	364/1212 (30%)	1212	Active	No treatment	2SHRZ 3SHRZ 3SPH / 9S_2_H_2_	Clinical, radiological, or bacteriological diagnosis of TB at 5 years
Anastasatu (1985) [35]	Hospital setting (not reported)	Romania	230/634 (36·3%)	634	Active	No treatment	3H_2_S_2_E_2_ 3H_2_S_2_Z_2_ 3R_2_H_2_S_2_Z_2_	Bacteriological diagnosis of TB at 2 years
Cowie (1985) [37]	CXR surveillance in a goldmine (ACF)	South Africa	Not reported	402	Active	No treatment	3RHZE	Bacteriological or histological diagnosis of TB at 5 years
Anon (1989) [41]	Adults presenting to care (PCF)	Hong Kong	592/1959 (30·2%)	1959	Active	No control arm	3SHRZ 3S_3_H_3_R_3_Z_3_ 4S_3_H_3_R_3_Z_3_	Clinical, radiological, or bacteriological diagnosis of TB at 5 years
Norregaard (1990) [32]	Adults presenting to care (PCF)	Denmark	22/72 (30·6%)	72	Active	No treatment	3RHE / 6HE	Bacteriological diagnosis of TB during follow up of at least 3 years
Teo (2002) [34]	Adults presenting to care (PCF)	Singapore	113/314 (36%)	314	Active	No control arm	2HRZ / 2HR 2HRZ / 2H_3_R_3_	Clinical, radiological, or bacteriological diagnosis of TB at 5 years
Ohmori (2002) [30]	Annual CXR surveillance (ACF)	Japan	0/29 (0%)	29	Inactive	No treatment	6H	Clinical, radiological, or bacteriological diagnosis of TB at 5 years
Turkova (2022) [11]	Symptomatic children presenting to care (PCF)	Uganda, Zambia, South Africa, India	165/1204 (14%)***	1204	Active	No control arm	2HRZ(E) / 2HR 2HRZ(E) / 4HR	Unfavourable status (treatment failure, treatment extension, TB recurrence, loss to follow-up, death) at 72 weeks

*Disease activity (active | inactive | mix) as determined by study radiologist. Inactive changes typically described as fibrotic or calcified only.

**Could be extended per clinician judgement

***Culture or Xpert MTB/RIF positive

RCT = Randomised controlled trial, ACF = Active Case Finding, PCF = Passive Case Finding, H = Isoniazid, PAS = p-aminosalicyclic acid, Th = thioacetone, S = streptomycin, R = rifampicin, Z = pyrazinamide, E = ethambutol. Numbers prior to drugs denote number of months the regimen is given for. Numbers in subscript after a drug denote how many days a week it is administered on if not daily. E.g. 6HPAS indicates 6 months of daily Isoniazid and p-aminosalicyclic acid

Apart from Turkova *et al*., few manuscripts explicitly stated the primary outcome; however, where available, this was a diagnosis of active pulmonary TB by end of follow-up, which was at least five years for most studies. Five studies required bacteriological confirmation to meet their primary outcome of active TB, with the remainder permitting clinical or radiographic (progressive changes) diagnosis. Respiratory sampling was predominantly from sputa, but some studies permitted supplementary use of laryngeal swabs or gastric lavage.

Most studies included more men than women. Turkova *et al*. studied only children under 16 years old, whilst the rest studied adults. Although nine studies recruited participants over 15 years old, the study-level median age was generally not reported nor calculable (Supplementary Table S5 in S1 File). The process for radiographic description was clearly documented in 10/13 studies, and was usually a panel of clinicians or two independent radiologists. There were no specific definitions presented for what constituted “active” or “inactive” TB radiographically. The reporting of baseline CXR changes varied: four studies reported on the presence of bilateral changes (range 6–26%) and five reported on the presence of cavities (range 1–24%). Most studies described the CXR changes as “active”, except for Ohmori *et al*. and Thompson *et al*., which required stable, fibrotic changes (e.g. “inactive” TB) as an inclusion criteria. Four studies radiographically monitored participants for up to twelve months prior to recruitment to ensure changes were not progressing. For studies involving only those with active CXR changes, there appeared to be a correlation between case-finding methodology and rate of sputum culture positivity at baseline, with higher rates of culture positivity in studies using PCF (30–36%) compared to ACF (5–7%); there was no impact from the number of sputum samples collected at baseline. There was minimal information on symptomatology at baseline. Apart from Turkova *et al*., no study clearly reported on the proportion of participants with previous TB, nor participant HIV status, although the majority were conducted prior to, or early in, the global HIV epidemic [20]. Only Ohmori *et al*. and Norregaard *et al*. reported on the background incidence rate of TB and most studies were conducted before TB incidence data was routinely available from the WHO.

Several treatments were evaluated and after qualitative analysis these were grouped into two categories: isoniazid-based, and multi-drug. Six studies, including the oldest five (1963 to 1982) used isoniazid-based regimens: isoniazid with or without a weak partner agent (e.g. thioacetazone, para-aminosalicylic acid) given for 3–12 months, akin to regimens used for latent TB infection. The remaining seven studies used multi-drug regimens: three or more potent agents including isoniazid and rifampicin and/or streptomycin, mostly administered for 2–3 months. Where reported, dosages of isoniazid and rifampicin were similar across trials.

Seven studies were excluded from the meta-analysis due to insufficient sampling and reporting of culture outcome data (Clayson *et al*.), absence of an inactive comparator (Anon (1989), Teo *et al*., Turkova *et al*., Anastasatu *et al*.), and for only including participants with solely fibrotic CXR changes (Thompson *et al*., Ohmori *et al*.) (Table 1). For the remaining six studies, pooled analysis found progression to bacteriological-confirmed pulmonary TB was significantly reduced with treatment compared to no treatment (risk ratio [RR] 0·27, 95% CI 0·13–0·56, I^2^ = 90·4%) (Table 2).

**Table 2:** Study results.

	Follow up time (years)	Control Arm		Intervention Arm(s)		Unadjusted RR (95% CI)
		Control	Risk of incident TB n/N (%)	Intervention	Risk of incident TB n/N (%)	
Studies included in pooled analysis						
Frimodt-Moller (1960) [36]	Not reported	Placebo	26/86 (30.2%)	3H (+-PAS)	28/89 (31.5%)	1.04 (0.55–1.96)
				6H (+-PAS)	30/83 (36.1%)	1.20 (0.64–2.23)
				12H (+-PAS)	17/91 (18.7%)	0.62 (0.31–1.25)
Pamra (1971) [39]	6	Placebo	57/178 (32.0%)	12H	10/139 (7.2%)	0.22 (0.12–0.42)
Aneja (1979) [38]	1	Placebo	21/110 (19.1%)	12HTh	11/103 (10.7%)	0.56 (0.28–1.10)
Anon (1984) [40]	5	No treatment	71/173 (39.9%)	2SHRZ	10/161 (6.2%)	0.15 (0.08–0.30)
				3SHRZ	5/161 (3.1%)	0.08 (0.03–0.19)
				3SPH/9S_2_H_2_	1/160 (0.6%)	0.02 (0.00–0.11)
Cowie (1985) [37]	5	No treatment	88/152 (57.9%)	3HRZE	30/250 (1.2%)	0.21 (0.14–0.30)
Norregaard (1990) [32]	3	No treatment	8/28 (28.6%)	3HRE/6HE	0/22 (0%)	0.07 (0.00–1.23)
Studies not included in pooled analysis						
Thompson (1982) [31]	5	Placebo	97/6990 (1.4%)	3H	76/6956 (1.1%)	0.79 (0.52–1.18)
				6H	34/6965 (0.5%)	0.35 (0.22–0.57)
				12H	24/6919 (0.3%)	0.25 (0.15–0.42)
Ohmori (2002) [30]	2.5	No treatment	0/15 (0%)	6H	0/14 (0%)	1.07 (0.02–50.43)
Anon (1989) [41]	5	NA	NA	3SHRZ	10/389 (2.6%)	NA due to study design
				3S_3_H_3_R_3_Z_3_	10/370 (2.7%)	NA due to study design
				4S_3_H_3_R_3_Z_3_	4/359 (1.1%)	NA due to study design
Teo (2002) [34]	5	NA	NA	2HRZ/2HR	0/99 (0%)	NA due to study design
				2HRZ/2H_3_R_3_	1/102 (1.0%)	NA due to study design
Turkova (2022) [11]	1.5	NA	NA	2HRZ(E)/2HR	1/332 (0.3%)	NA due to study design
				2HRZ(E)/4HR	0/335 (0%)	NA due to study design
Anastasatu (1985) [35]	2	Adjacent cohort**	6/143 (4.2%)	3H_3_S_3_E_3_	5/68 (7.4%)	NA due to study design
				3H_2_S_2_Z_2_	4/60 (6.7%)	NA due to study design
				3R_2_H_2_S_2_Z_2_	2/76 (2.6%)	NA due to study design

NA = Not applicable

*Clayson (1963) [33] not included as no sputum culture data available

**This arm was not a randomised comparison, but rather lower-risk participants selected for observation without treatment

**Fig 2:**
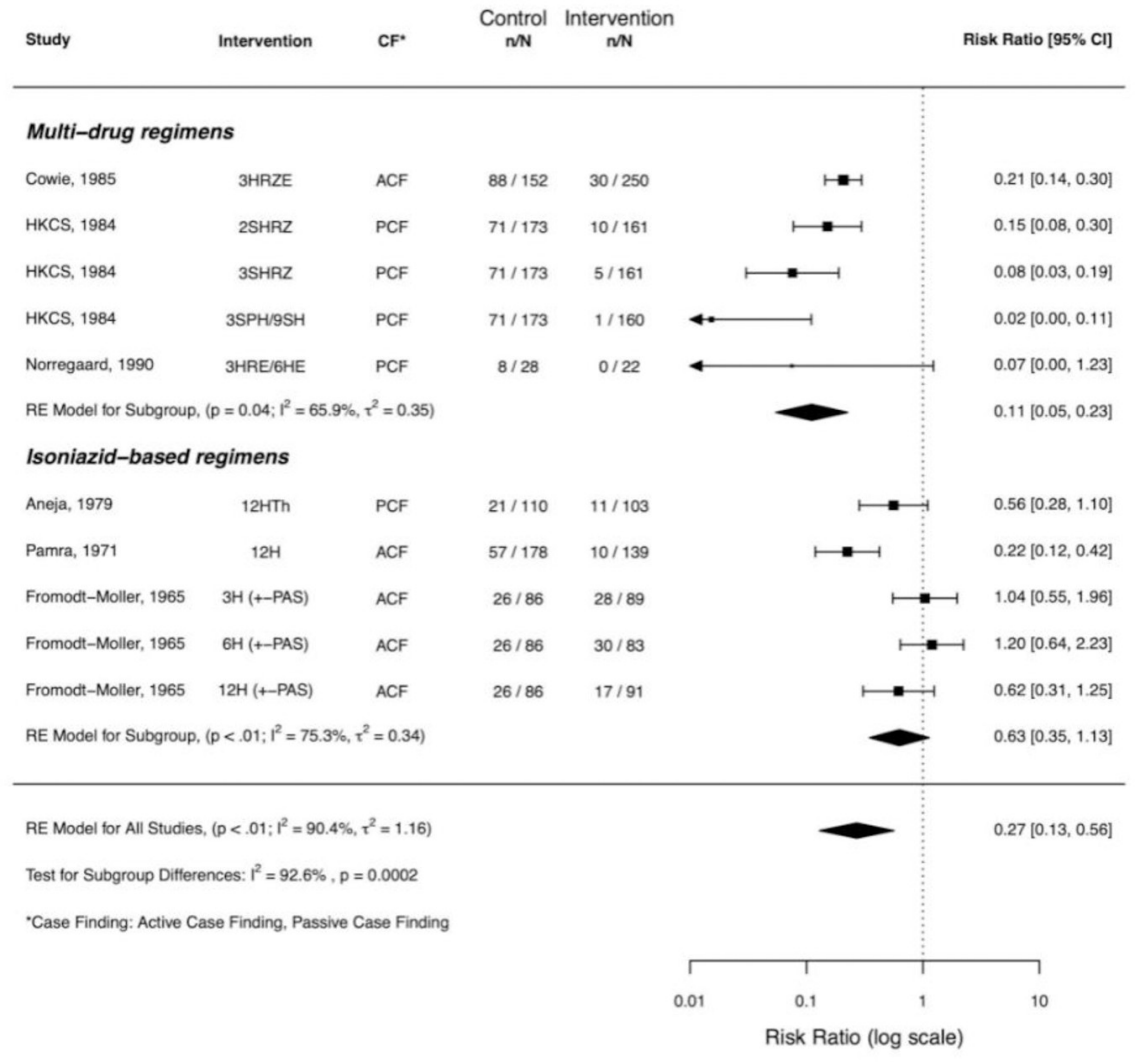
Forest plot for treatment results by regimen.

Subgroup analysis by multi-drug vs isoniazid-based regimens found a significant difference in treatment effect (p<0·001) and reduced proportion of variability due to heterogeneity (I^2^ = 65·9% and 75·3% respectively). Multi-drug regimens conferred greater risk reduction (RR 0·11, 95% CI 0·05–0·23) in comparison to isoniazid-based regimens, which showed no conclusive benefit (RR 0·63, 95% CI 0·35–1·13) (Fig 2). This effect remained unchanged in a sensitivity analysis using only regimens containing rifampicin (RR 0·15, 95% CI 0·09–0·25). The NNT for multi-drug regimens was 2·5 (95% CI 2·2–3·0). Other subgroup analyses were statistically significant, with the intervention arm yielding a larger risk reduction in studies with PCF methodology compared to ACF (p = 0·037), and where more respiratory samples were collected at baseline (p = 0·018) (Supplementary Figures S7, S8 in S1 File). However, on review of the data and degree of impact on heterogeneity, these findings appeared to be confounded by regimen type. There was insufficient data to support other planned subgroup analyses (HIV status, radiographic change at baseline).

Although we had planned to explore other markers of TB disease progression or regression (e.g. clinical or radiographic), no studies presented sufficient data. Reporting on rates of loss to follow-up was variable, and for studies where it was clearly presented it ranged widely (0 to 17·5%). Three studies reported on adherence, with two finding a negative correlation with duration of therapy—the percentage of people taking most of their medication was higher in those allocated 6-months treatment compared to 12-months: 87% vs 68% in Thompson *et al*., and 63% vs 25% in Frimodt-Moller *et al*. [36].

Reporting of safety outcomes was limited and inconsistent. Two studies reported that 2–6% of participants had at least one drug stopped. Cowie *et al*. reported no reactions in 250 patients receiving three months of HRZE (isoniazid, rifampicin, pyrazinamide, and ethambutol), and Clayson *et al*. reported 3/94 participants experiencing severe reactions whilst receiving a six-month regimen of H-PAS (isoniazid and para-aminosalicyclic acid). Drug resistance was described in seven studies, there was substantial variation in reporting, no description of laboratory methodology, and testing was often done on a small subset. For most studies using multi-drug regimens, the rates of resistance were low (0–2%). Two studies using isoniazid-based regimens reported high rates of resistance (15–47%), but it was not possible to ascertain if there was a difference between the intervention and control arms. No study reported any patient-centred outcomes (e.g. quality of life, catastrophic costs) or recorded patient perspectives of TB treatment. Turkova *et al*. reported a cost-effectiveness analysis finding reduced health care costs for those given a shorter regimen.

**Fig 3:**
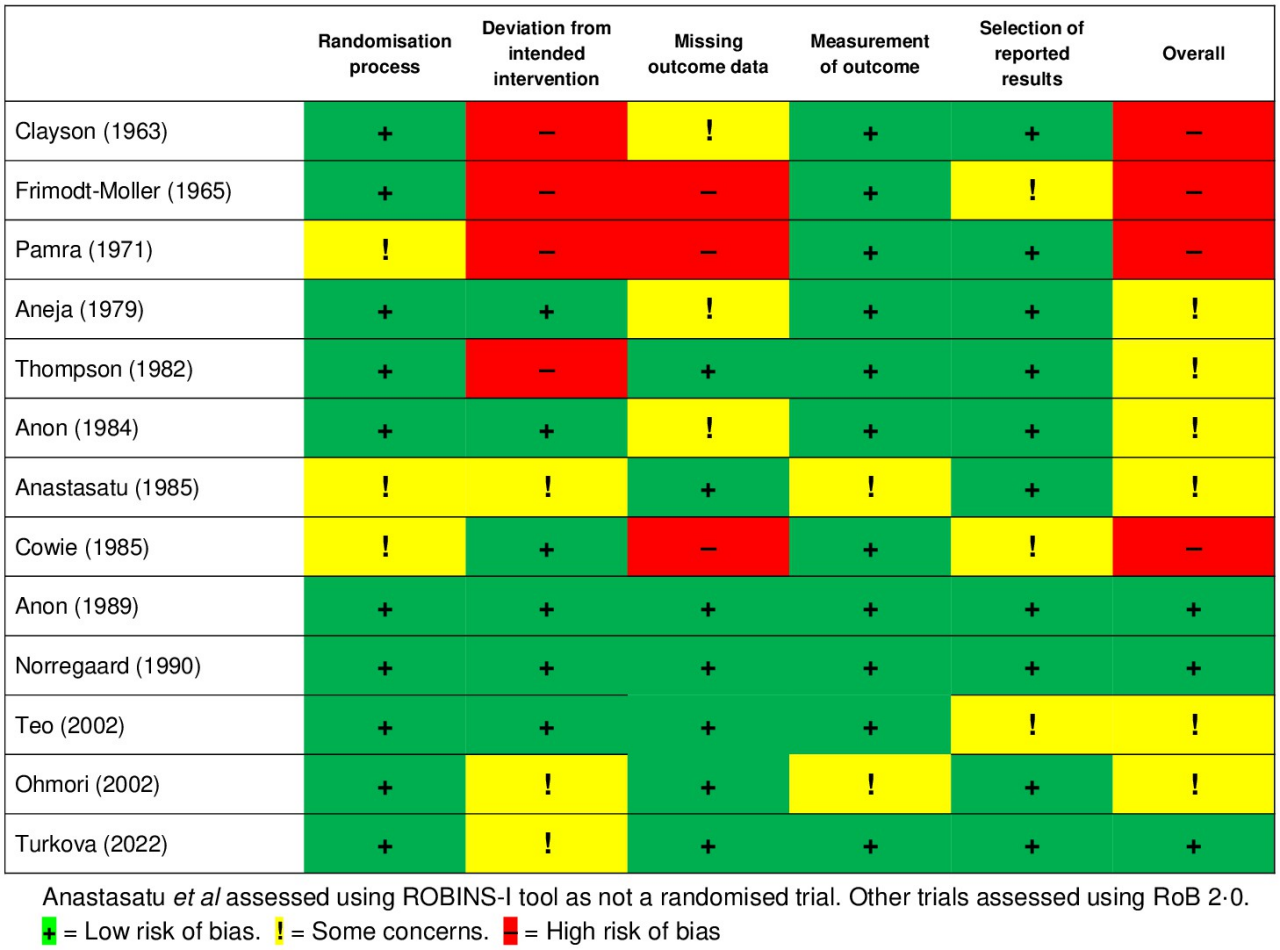
Quality assessment of included studies.

Overall, the quality of included studies was low, with the oldest studies assessed as at high risk of bias (Fig 3). Reporting of study methodology was often not standardised making it unclear whether there had been deviation from the intended intervention, or how the randomisation had been administered, and it was frequently not possible to assess the primary outcome at multiple time points or to assess secondary outcomes. Only Aneja *et al*. reported using a double-blinded placebo. The funnel plot was asymmetrical, implying presence of publication bias (Supplementary Figure S6 in S1 File).

Sensitivity analyses, firstly excluding studies with high risk of bias (leaving 3 studies), and where primary outcome included TB diagnosis by clinical/radiological progression (7 studies), found comparable estimates of effect to our primary analysis (Supplementary Table S9 in S1 File). A network analysis including two studies with comparable intervention arms found that longer duration of treatment was associated with improved outcomes (Supplementary Table S10 in S1 File).

## Discussion

This systematic review identified 13 trials in adults and children with CXR features of TB and negative sputum cultures, conducted between 1955 and 2018 in a variety of settings. The risk of progression from culture-negative to culture-positive TB for individuals with active CXR changes but no treatment was substantial (19–58%), and meta-analysis of six studies showed that treatment can reduce this risk (RR 0·27, 95% CI 0·13–0·56). For those treated with a multi-drug regimen, the rate was substantially and significantly lower compared to placebo or observation (RR 0·11, 95% CI 0·05–0·23), and although there was potentially an impact from duration, regimens of 2–3 months appeared to offer excellent results. Although we acknowledge limitations of pooled NNT calculations, a NNT of 2·5 is substantially lower than estimates for treatment of latent tuberculosis infection (NNT of 100) [21]. Studies using isoniazid-based regimens did not show the same impact of treatment (0·63, 95% CI 0·35–1·13).

Current guidelines on the management of this group of patients is variable and non-specific. A trial of antibiotics is frequently recommended [22], though recent evidence refutes the evidence base for this approach highlighting the potential harm of delayed treatment [23]. The Centres for Disease Control and Prevention / Infectious Diseases Society of America guidelines support the use of a four-month regimen based on the findings from the Hong Kong studies [24], however, implementation of this in practice is low. Most existing guidelines have not incorporated evidence from the studies included in this review, which is unsurprising as the regimens evaluated are largely outdated, and the described phenotype (CXR positive, culture-negative) is not officially recognised. WHO guidelines for management of latent TB infection suggest that if an individual with CXR changes has active TB excluded bacteriologically, preventive therapy could be considered. The results of this review provide evidence to suggest that IPT (isoniazid preventive therapy), particularly if given for only six months, would not be appropriate for patients with CXR changes suggestive of active TB, even if sputum culture is negative. There is currently little evidence to support the use of other preventive regimens for this group; however, experience of treating with four months of rifampicin and isoniazid in an observational cohort of 414 patients with CXR suggestive of active TB with three negative sputum cultures found very low rates of relapse (1·2% culture-positive (all drug-sensitive) over 78 months), although this regimen is yet to be evaluated in a clinical trial [25]. All included studies were conducted prior to or early on in the global HIV epidemic, with no discussion of recurrent TB infections and its relevance to chronic CXR changes, and before the introduction of molecular diagnostics. False positive sputum PCR results (PCR positive, culture-negative) have been described several years after successful treatment completion and their relevance is poorly understood [26].

Our findings add weight to the idea that patients in the middle of the TB disease spectrum are distinct to patients with sputum culture-positive disease or latent TB infection, and should be managed differently [9]. Given the rapid expansion of CXR-based screening programmes globally, this group of patients will be increasingly identified. Our review highlights the need for clinical trials to identify appropriate diagnostic and treatment options, and may have broader relevance for the development of trials hoping to incorporate novel biomarkers (e.g. blood transcriptomics) and advanced radiological methods (e.g. positron emission tomography / computed tomography) which may better identify patients at increased risk of disease progression [27].

This review has limitations. The number of identified studies was small, some of which had low participation, and there was significant between-study heterogeneity. A variety of regimens and durations were used, many of which contained drugs now rarely used. However, we were able to broadly categorise treatments into isoniazid-based and multi-drug, which provided valuable insights. We were not able to explore differences in treatment effects by symptomatology due to limited reporting and confounding factors. We recognise that variation in follow-up and missing baseline, outcome, and safety data could have introduced reporting bias, although sensitivity analyses showed no change in treatment effect. Missing or incomparable data due to heterogeneity of study methodology meant several pre-defined sub-group analyses were not possible. Most studies were conducted over 25 years ago and there have been advances in the diagnostic approach to TB with respect to imaging (e.g. digital x-ray, computer-aided-detection) and microbiological techniques (e.g. molecular diagnostics, liquid culture), likely improving sensitivity and specificity. Furthermore, laboratories would have been less well equipped by modern standards, with limited or no quality assurance, potentially leading to misclassification of the primary outcome by either under or over-reporting. Laboratory capability to observe for acquired drug resistance was also limited. The lack of presented data on specific participant comorbidities (e.g. diabetes, HIV) limits the applicability of findings to these groups. Six studies excluded participants with prior TB history, increasing the probability that CXR changes were due to active disease, but reducing the generalisability of our results.

Rifapentine-based regimens have recently been shown to reduce the required treatment duration for culture-positive disease and latent TB infection to four months and one month respectively [28, 29], however, none of the regimens evaluated in this review contained rifapentine.

Despite limitations, this review finds that abbreviated, multi-drug regimens for culture-negative pulmonary TB can prevent progression to culture-positive disease. Isoniazid, particularly if used for only six months, appears unlikely to prevent progression. This study population is becoming increasingly relevant as CXR-based screening expands and diagnostic tests with the potential to identify incipient TB become available. Modern clinical trials are necessary in order to establish the optimal approach to treatment in this patient group as we re-define the previously accepted binary approach to TB management.

## Supporting information

S1 File Supplementary materials. (DOCX)

S2 File Supporting data. (XLSX)

S1 Checklist PRISMA 2020 checklist. (DOCX)

S2 Checklist PRISMA 2020 for abstracts checklist. (DOCX)
